# Influence of Incorporated Polydimethylsiloxane on Properties of PA66 Fiber and Its Fabric Performance

**DOI:** 10.3390/polym11111735

**Published:** 2019-10-23

**Authors:** Wei Peng, Ying Qian, Tong Zhou, Shenglin Yang, Junhong Jin, Guang Li

**Affiliations:** State Key Laboratory for Modification of Chemical Fibers and Polymer Materials, College of Materials Science and Engineering, Donghua University, Shanghai 201620, China; pwpz321654@hotmail.com (W.P.); viskyying@163.com (Y.Q.); 15921876270@163.com (T.Z.); slyang@dhu.edu.cn (S.Y.); jhkin@dhu.edu.cn (J.J.)

**Keywords:** poly(hexamethyllene adipamide), polydimethylsiloxane, blending fiber, fabric performance

## Abstract

Poly(hexamethyllene adipamide), PA66 fiber has played an important role in varied industrial applications, and its corresponding product would become more competitive if some extra value was added to PA66 fiber. In this article, polydimethylsiloxane (PDMS) was used as an additive to prepare PA66/PDMS blend fibers through melt blend spinning carried out by a screw extruder spinning machine. When the amount of incorporated PDMS was 0.5–3 wt %, the blend melt demonstrated good spinning ability, and the PA66/PDMS blend fibers exhibited excellent mechanical property and reduced hot shrinkage. Moreover, the crystallization and melting behavior of PA66 in the blend fibers turned out to be not affected by the existence of PDMS. In addition, the contact angle of water on the blend fiber surface became larger, while the value of friction coefficient on the surface of fibers got lower with increasing PDMS content in the blend fibers. After evaluating the fabric woven by PA66/PDMS blend fibers using the KES-F KES-FB-2 fabric measuring system, it was found that as PDMS content increased, the flexural rigidity and bending hysteresis would be lower, yet elasticity rate of compression work would be higher, which explained how the fabric composed of the blend fiber performed better in terms of softness and elasticity.

## 1. Introduction

Polydimethylsiloxane (PDMS), commonly known as silicone, has been generally used as an additive in polymer processing [1,2]. PDMS can improve flow-ability of melt which is especially required under some circumstances such as processing some products with thin walls and complex shapes [3], where full filling of injected melt is needed. PDMS can also reduce friction between products and equipment [4], keeping the surface of products bright and clean to make product quality high and more competitive.

Hager et al. [5] prepared a multifunctional additive based on PDMS of ultrahigh molecular weight. He found this additive highly effective regarding mobility of thermoplastic processing, especially applicable to polyolefin processing. Besides, this additive not only strengthened impact and tensile strength of products, but made the surface highly polished and resistant to abrasion as well. Jin et al. [6] prepared a long branched PDMS grafted on PE chains (PDMS–*g*–PE) and used it as plasticizer to improve the melt processing of HDPE high-density polyethylene (HDPE). It was discovered that melt flow index would decrease when PDMS-g-PE content was 2 wt %, suggesting melt mobility of HDPE was improved to a certain extent. In practical use, PDMS has also been commonly used to coat fibers and fabrics, acting as an oil or finishing agent to make fiber and its product have a low friction coefficient and a smooth feeling [7,8,9]. At the same time, it was noticed that PDMS on the surface can be easily washed away during the dyeing and washing process, which would not only invalidate the function of PDMS itself, but also bring in negative consequences to the environment.

The purpose of this study is to incorporate PDMS into PA66 during the melt-blend spinning process through a screw extrude machine. Thus, PDMS can be distributed both inside and on the surface of PA66 fiber, leading to low friction on the fiber surface. Moreover, PDMS will move onto the surface gradually in practical use, finally realizing the enrichment of PDMS on fiber surface. As a result, the surface friction coefficient could be reduced and the water contact angle on fiber surface could be enlarged, in addition, the fibrous fabric would become soft and fluffy with function of waterproofing and antifouling as well due to the presence of PDMS.

## 2. Experimental

### 2.1. Materials

PA66 chips were from Shenma Group, Pindingshan, China, FYR-27, PDMS master-batch, MB50011, from Dow Corning Ltd. (Soochow, China).

### 2.2. Preparation of PA66/PDMS Blend Fibers

Blend chips of PA66 with addition of PDMS (0.5–3.0 wt %) were dried in vacuum at 110 °C for 48 h. Then, PA66/PDMS blend fibers were obtained by melt-blend spinning using ABE-25 spinning machine. Each as-spun fiber was received at 1000 m/min of take-up speed, and then stretched out using through TF-100 stretching equipment with heat board at 60 °C and heat plate at 120 °C, with drawing ratio of 3.5, 3.7, 3.9, and 4.1, respectively.

### 2.3. Methods

#### 2.3.1. Capillary Rheological Performance Measurement of PA66/PDMS Blends

First, PA66/PDMS blend chips were obtained through melt-mixing by twin-screw extruder where PA66 and PDMS were dried at 110 °C for 48 h to remove water in advance. The capillary rheological behavior was investigated using Marrin RH2000 Capillary Rheometer at 275, 280, and 285 °C, respectively, with a capillary radius of 0.5 mm, L/D ratio 16:1, and sheer rate in the range of 100–10,000 s^−1^.

#### 2.3.2. Differential Scanning Calorimetry (DSC)

DSC spectra were recorded on TA-Q20 DSC apparatus (city, country Shanghai, China). In the test, 5–10 mg dried samples were first heated to 300 °C, kept for 3 min at that temperature, then cooled down to room temperature at a rate of 10 °C/min. Finally, the samples were heated again to 300 °C at the same rate of 10 °C/min.

#### 2.3.3. Thermal Shrinkage

Thermal shrinkage of PA66/PDMS blend fibers was measured manually according to GB/T6505-2001. Before testing, fibers were balanced at standard environment for 3 h; next they were treated in an oven at 190 °C for 15 min, and then balanced for 4 h. Every sample was examined three times to averagely calculate thermal shrinkage as the formula: S = (L_0_ − Ls)/L_0_ × 100%, where S is thermal shrinkage (%), L_0_ is the original length of fiber (mm), Ls is the length after heating treatment (mm).

#### 2.3.4. Preparation of PA66/PDMS Blend Fibrous Fabric

PA66/PDMS blend fibers containing different ratio of PDMS with the same drawing ratio of 3.5 were woven into simple plain texture fabric, respectively, in handed shuttle loom in our lab. Each fabric was woven at the same condition by manual control as best as one can.

#### 2.3.5. Silicon Analysis by Energy Dispersive Spectra-Meter (EDS)

PA66/PDMS blend fibers were washed by acetone and distilled water to remove oil on the surface, then dried and kept under constant temperature of 20 °C and humidity of 65% prior to test. With help from IncaX-Max EDS (Hitachi G, Japan), the presence of PDMS (Si) and its distribution was investigated on cross section and the surface of fibers.

#### 2.3.6. Water Contact Angle on Fiber

The German OCA40Micro optical contact angle measuring device (Germany) was used. Before the experiment, the blend fiber was washed by acetone and distilled water for surface degreasing, and then drying and balancing under temperature of 20 °C and relative humidity of 65%. During testing, the volume of water drop was controlled at 300 μL and 0.65 Pa. The average value was taken from 30 test results for each sample.

#### 2.3.7. Friction Coefficient of Fiber Surface

XCF-1A fiber friction meter (mode: friction roller rotation) was employed for measurement of friction coefficient of fiber surface. The blend fiber was washed by acetone and distilled water, then dried and kept under constant temperature of 20 °C and humidity of 65% prior to the test. The pre-tension was set as 0.1 cN, rotation speed 30 rpm, and decline rate of friction roller 10 mm/min. The average value was taken based on 15 test runnings for each sample.

#### 2.3.8. Bending Properties of Fabrics

According to GB/T 18318.5-2009, KES-FB-2 equipment was used to examine bending properties of fabrics, from which flexural rigidity (B) and bending hysteresis (2HB) could be obtained for further analysis. Before the test, the standard temperature and humidity conditioning were implemented in accordance with GB/T 6529.

#### 2.3.9. Compression Performance of Fabrics

As instructions specified in GB/T 24442.2-2009 (part 2), compression performance was tested by KES-FB-3 equipment (city, countryShanghai, China) to obtain compression work (WC) and elasticity rate of compression work (RC). Temperature and humidity conditioning were implemented in accordance with GB/T 6529 before the test.

## 3. Results and Discussion

### 3.1. Effects of PDMS on Flowing Behavior of PA66 Melt

**Figure 1:**
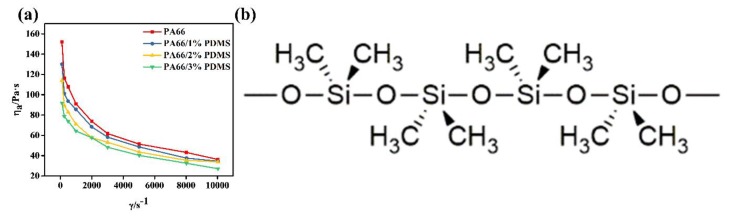
The flow curves of PA66 and PA66/PDMS melts (**a**), and chemical conformation of PDMS (**b**).

High melt flow ability was preferred during the fiber fabrication. When PDMS was added into PA66, the blend melt behavior was investigated using capillary rheometer. As illustrated in Figure 1a, the fluid curves of pristine PA66 and three PA66/PDMS mixed melts with addition of 1, 2, and 3 wt % PDMS are present. Similar to PA66, the apparent viscosity of mixed melts falls as shear rate increases; besides, the decreased extent is positively correlated with the content of PDMS added, suggesting a positive effect on flow ability of PDMS addition. This may be attributed to the mutual repulsion between Si–O and methyl groups in PDMS chains. As shown in Figure 1b, the outward arrangement of methyl groups, acting as a screen, can result in low surface energy and low surface tension. Thus, it can be seen as a lubricator reducing the force and entanglement density among PA66 molecular chains, and further reducing the apparent viscosity of the system. Therefore, the addition of certain amounts of PDMS to PA66 is able to improve melt flow ability and help the formation of PA66 fiber.

### 3.2. Thermal Properties of PA66/PDMS

**Figure 2:**
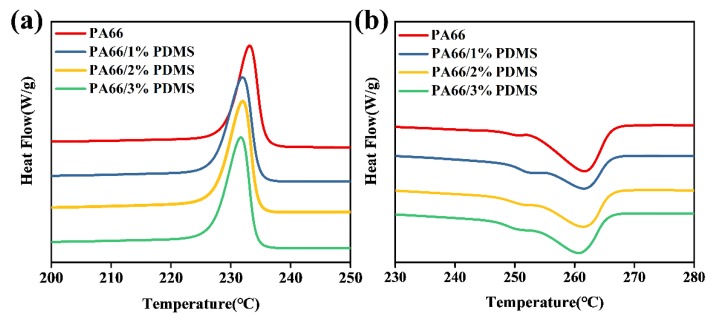
Crystallization (**a**) and melting (**b**) of PA66/PDMS blends.

Figure 2 exhibits the crystallization and melting curves of the mixed PA66/PDMS melt with different PDMS contents, from which no significant effect is observed on crystallization and melting of PA66 coming from PDMS addition. Crystallization temperature of PA66 stabilized at 232–233 °C, and the melting point of PA66 stayed at 262–263 °C. The existence of PDMS is able to enhance mobility of the melt, and at the same time gives it good thermostability and does not influence crystallization and fusion of PA66. This can be beneficial to formation and application of modified PA66 fibers.

### 3.3. Mechanical Properties of PA66/PDMS Blend Fibers

**Figure 3:**
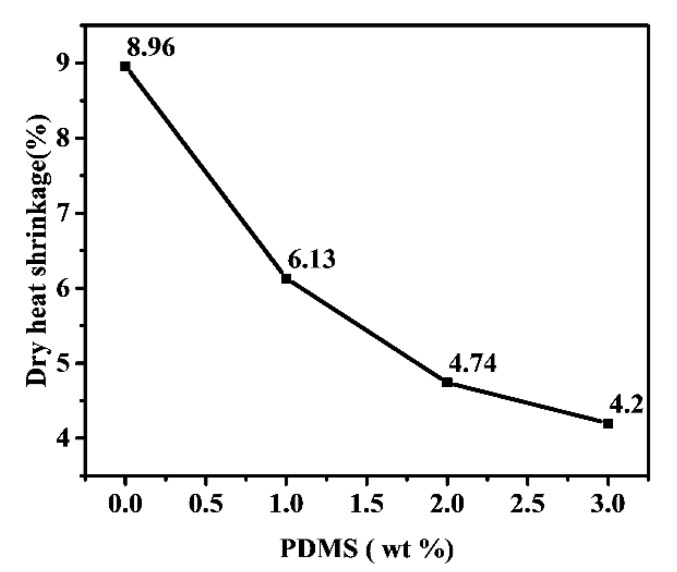
Correlation between dry heat shrinkage and PDMS content.

The PA66/PDMS blends with 0.5–3.0 wt % of PDMS could be melt-spun into fibers well even when the melt temperature has a drop of 2–3 °C. Especially when PDMS addition is 0.5–1.0 wt %, the blend showed outstanding spinning capability. Furthermore, when comparing the mechanical properties among all obtained fibers, the addition of 0.5–1.0 wt % PDMS was found optimal. As listed in Table 1, certain enhancement of tensile-strength was observed, which may be ascribed to the role of PDMS that could improve mobility of PA66 chains, lowering entanglement of PA66 macromolecular chains. Under the same drawing ratio, the existence of PDMS can make the blend fiber maintain a high elongation at break, which could lead to a better hand feeling of fiber and textile. Regarding dry heat shrinkage, the test results (Figure 3) gave a declining trend when more PDMS was added, indicating a better performance of dimensional stability under high temperature. The reason for this reduced thermal shrinkage may be ascribed to the presence of PDMS which could decrease intermolecular interaction and internal stress in fiber.

### 3.4. Surface Properties of PA66/PDMS Blend Fibers

**Figure 4:**
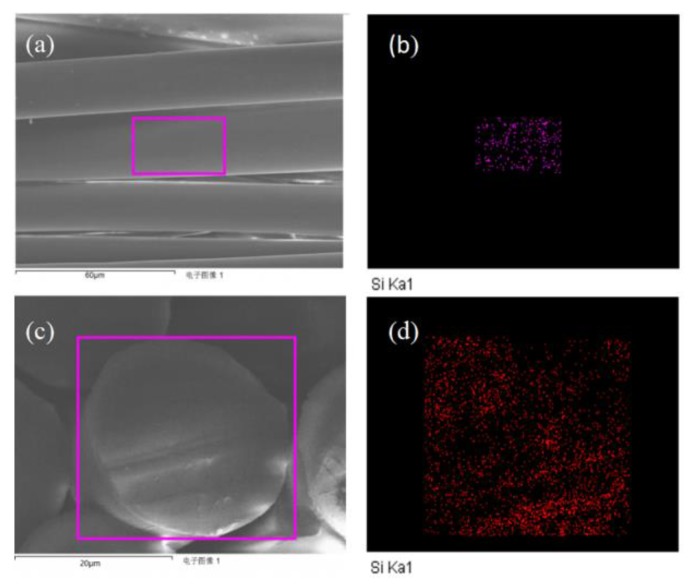
Electron scanning micrograph (**a**,**c**) and element mapping of surface and cross section of PA66/3%PDMS blend fiber (**b**,**d**).

EDS was applied to detect whether silicon existed on the surface or cross section of PA66/PDMS blend fibers. Figure 4a,c illustrates the surface and cross section morphology of PA66/PDMS (3%) blend fiber with 3 wt % of PDMS, while its corresponding silicon mapping can be found on Figure 4b,d. It is noticed that silicon exists on both surface and cross section of the fiber, also there is a trend showing silicon moving onto the surface. Apart from that, the element content analysis explains that the amount on the surface is higher than inside, ensuring when external silicon is falling off as time passes, the element within can still move and concentrate onto the outer layer. This, in contrast with other surface coating or fishing with silicon agent, could function well in long duration as well.

The existence of silicon on the surface could decrease the value of surface friction coefficient. According to Table 2, the dynamic (µs) and static (µd) friction coefficient of pristine PA66 is 0.3712 and 0.3217, respectively. With the increasing addition of PDMS, both µs and µd experience a downward trend, e.g., comparing pristine PA66 fiber with PA66/1%PDMS fiber, there is a significant drop in both µs and µd values. This finding is consistent with the concentration of PDMS on the fiber surface which tends to become saturated with increasing PDMS addition. A small value of static and dynamic friction coefficient means better behavior in aspects of smoothness and stain-resistance of fibers.

**Figure 5:**
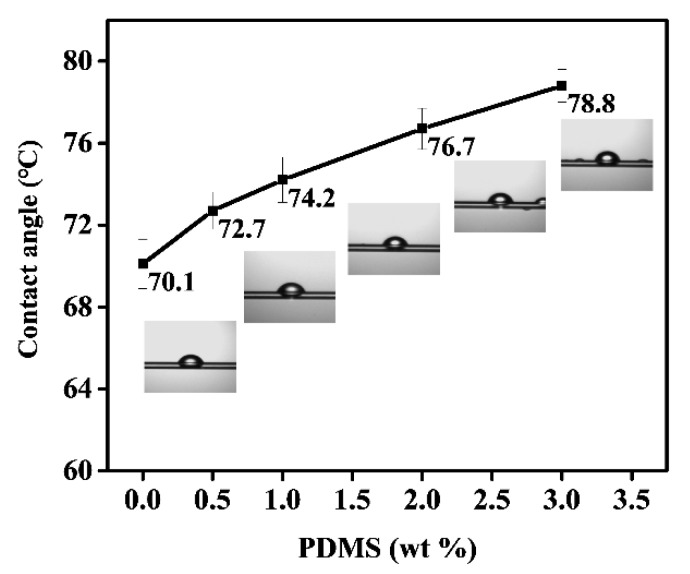
Correlation between PDMS content (%) and contact angle.

It was reported that when PDMS was used to treat cotton fiber and its fabric for surface coating the directional alignment of PDMS was observed, i.e., the polar silicon–oxygen bond pointed to the surface and the silicon-containing methyl to the air, which together formed a hydrophobic layer [1,10]. We can expect similar results emerge in obtained PA66/PDMS blend fibers. The water contact angle on fiber surface was given in Figure 5, suggesting an increase of contact angle as PDMS increases. A large contact angle could be able to improve waterproof performance, which, together with help from anti-fouling property endowed by small friction coefficient, can add extra value to sportswear et al.

### 3.5. Bending Deformation Properties of PA66/PDMS Blend Fibrous Fabrics

**Figure 6:**
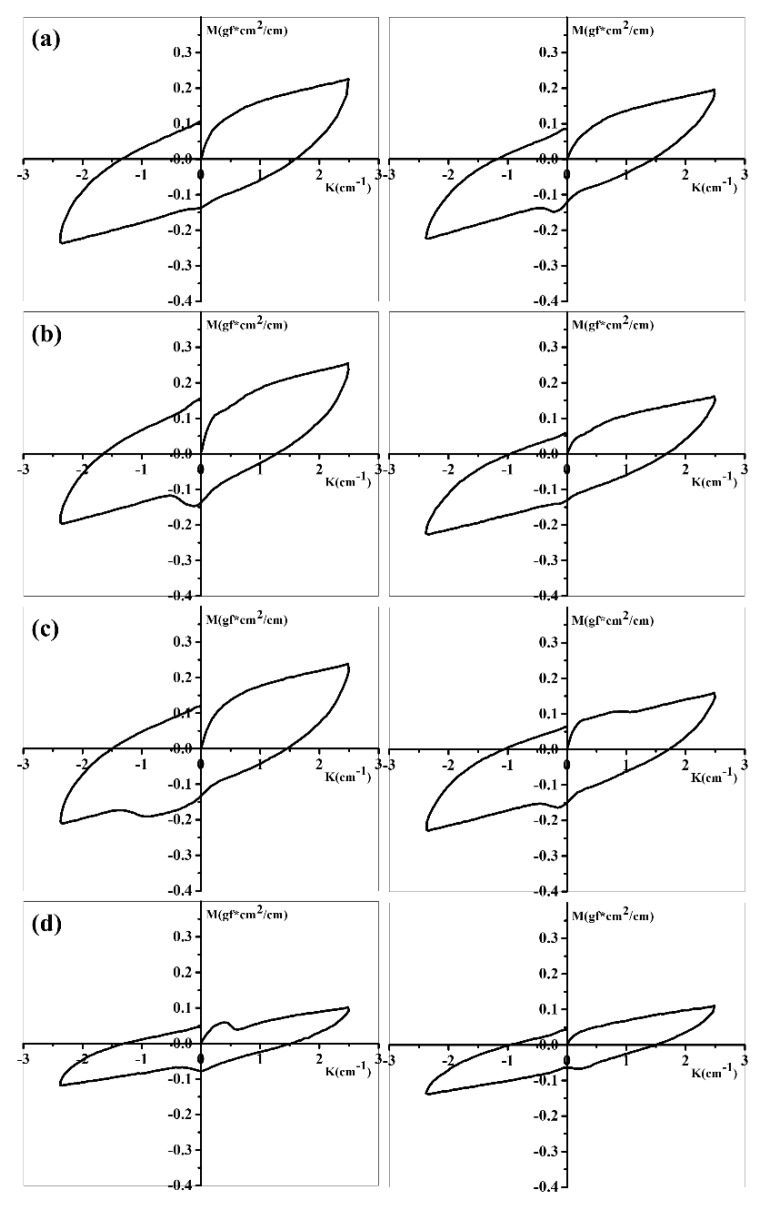
Bending deformation properties of the fabric: (**a**) PA66 in wrap and weft, (**b**) PA66/1%PDMS in wrap and weft, (**c**) PA66/2%PDMS in wrap and weft, (**d**) PA66/3%PDMS in warp and weft.

All fabric understands bending deformation during textile processing and practical application [11,12]. The resistance of fabric to this bending is defined as bending rigidity (B), i.e., the ratio of small variety of bending moment per unit width over the change in curvature in response. The smaller the value is, the softer the fabric would be. Combined with elasticity represented by 2HB called bending hysteresis, we can evaluate the general performance of hand feeling and fabric style. 2HB stands for the margin between the curvature per unit width when the sample is forced and the curvature value after recovery when the force is released. A smaller value of 2HB implies larger fabric elasticity and greater bouncing style. Figure 6 is the force–curvature curves of different fabrics, after calculation based on we can find that with incorporation of PDMS in fiber, the blend fibrous fabric exhibits declining tendency of B and 2HB in both wrap and weft (Table 3), suggesting an improvement in softness, elasticity, and bouncing style of fabrics.

Basically, the softness, elasticity, and bulkiness of fabric can be interpreted by compression properties in the direction of thickness, whose indexes can be listed as compression linearity (LC), compression work (WC), and elasticity rate of compression work (RC). With reference to Table 4, after addition of PDMS, LC and WC do not express significant change while RC experiences an increasing tendency as PDMS increases. RC represents the recovery ability after the pressure on fabric is released; the larger the value, the better the recovery ability the fabric has. Consistent with the bending deformation test above, the fibrous fabric performs better in elastic recovery properties with presence of PDMS in fiber.

## 4. Conclusions

PDMS can be successfully incorporated and well distributed in PA66 matrix fiber through a simple direct melt-mixing and spinning process. The obtained PA66/PDMS blend fiber showed satisfied mechanical properties and reduced thermal shrinkage at elevated temperature, while almost no effect on crystallization and melting behavior of PA66. Furthermore, with addition of PDMS, the decreased surface friction coefficient and increased water contact angle on the PA66/PDMS fiber surface was found, which may provide function of antifouling, waterproof, and stain-resistant ability of the fiber and its fabric. Fabric style measurement by KES serial equipment explained that PA66/PDMS blend fibrous fabric presents improved handing of smoothness, softness, and also a high level of elasticity against compression. Comparing with silicon compound coating or finishing on fiber and fabric surface, the method reported in this article is more eco-friendly and the results proved effective.

## Figures and Tables

**Table 1:** Tensile strength and elongation of PA66/PDMS blend fibers with different addition of PDMS at varied drawing ratio.

Sample	Drawing Ratio	Tensile Strength (cN/dtex)	Elongation (%)
PA66	3.5	4.3	28.5
PA66/0.5%PDMS	3.5	4.5	35.2
PA66/1%PDMS	3.5	4.4	36.9
PA66	3.7	4.5	26.9
PA66/0.5%PDMS	3.7	4.7	29.5
PA66/1%PDMS	3.7	4.7	30.4
PA66	3.9	5.3	22.1
PA66/0.5%PDMS	3.9	5.5	23.9
PA66/1%PDMS	3.9	5.4	24.4
PA66	4.1	5.6	18.6
PA66/0.5%PDMS	4.1	5.8	18.9
PA66/1%PDMS	4.1	5.7	19.1

**Table 2:** Surface friction coefficient of the obtained fibers.

Sample	fs/10-3cN	µs	fd/10-3cN	µd
PA66	137.5	0.3712	127.0	0.3217
PA66/1%PDMS	110.8	0.2572	105.9	0.2404
PA66/2%PDMS	101.8	0.2276	98.2	0.2155
PA66/3%PDMS	99.8	0.2203	96.8	0.2110

**Table 3:** Bending deformation properties of fabrics.

Sample	B (cN·cm^2^/cm)		2HB (cN·cm/cm)	
	Warp	Weft	Warp	Weft
PA66	0.0549	0.0496	0.2000	0.1656
PA66/1% PDMS	0.0571	0.0447	0.1967	0.1596
PA66/2% PDMS	0.0277	0.0322	0.2191	0.1741
PA66/3% PDMS	0.0283	0.0313	0.0872	0.0931

**Table 4:** Compression properties of the fabrics.

Sample	LC	WC (cN·cm/cm^2^)	RC (%)
PA66	0.28	0.17	29.4
PA66/1% PDMS	0.25	0.17	31.6
PA66/2% PDMS	0.26	0.15	30.7
PA66/3% PDMS	0.22	0.12	33.3
